# Does a selective non-peptide angiotensin II type 2 receptor agonist reduce post-infarction left ventricular remodeling?

**DOI:** 10.1186/1532-429X-11-S1-P29

**Published:** 2009-01-28

**Authors:** Alexander B Jehle, Yaqin Xu, Joseph D DiMaria, Frederick H Epstein, Brent A French, R Jack Roy, Robert M Carey, Christopher M Kramer

**Affiliations:** University of Virginia, Charlottesville, VA USA

**Keywords:** Cardiac Magnetic Resonance, Late Gadolinium Enhancement, Left Ventricular Mass, Total Left Ventricular, Baseline Left Ventricular

## Purpose

Cardiac overexpression of the angiotensin II type 2 receptor (AT_2_-R) attenuates left ventricular (LV) remodeling following myocardial infarction (MI). We hypothesized that the use of a novel non-peptide AT_2_-R agonist **Compound 21 (C21)** would significantly reduce adverse post-MI remodeling in mice as measured by cardiac magnetic resonance imaging (CMR). Such a compound could offer significant translational potential.

## Methods

Forty nine C57/BL6 mice underwent 60-minute occlusion of the left anterior descending artery, followed by reperfusion. Nine died early post-MI, leaving 40 for further study. Twenty three received 0.3 mg/Kg/day C21 SQ by Alzet minipump and 17 were untreated. CMR images were obtained at baseline and post-infarct (days 1 and 28) using a Clinscan 7 T MRI system (Siemens/Bruker). Using cine gradient echo black blood imaging, six to 8 continuous short-axis slices were acquired, each 1 mm thick, from the apex to the base. Images were both electrocardiographic and respiratory gated, with echo time of 1.9 ms, 15 degree flip angle and field of view 25.6 mm, giving a spatial resolution of 200 × 200 × 1000 μm^3^. The TR was adjusted continuously to obtain 16 equally spaced phases during each cardiac cycle. Three signal averages were used, resulting in an acquisition time of approximately 4 minutes per slice and total imaging time of 30 to 45 minutes per mouse. For infarct sizing on day 1 post-MI, late gadolinium enhancement was performed using an inversion recovery sequence with an initial 180 degree inversion pulse, TR of 5.4, TE of 0.67 ms, TI 500 ms, 20 degree flip angle and three averages. The FOV was 25.6 mm, with a spatial resolution of 200 × 200 × 1000 μm^3^. The ARGUS (Siemens Medical Systems) image analysis program was used to define LV end-diastolic (LVEDV) and systolic volumes (LVESV), stroke volume (SV), ejection fraction (LVEF) and LV mass at baseline and day 28 post-MI as well as infarct size on day 1 post-MI as % of total LV mass.

## Results

**Figure 1:**
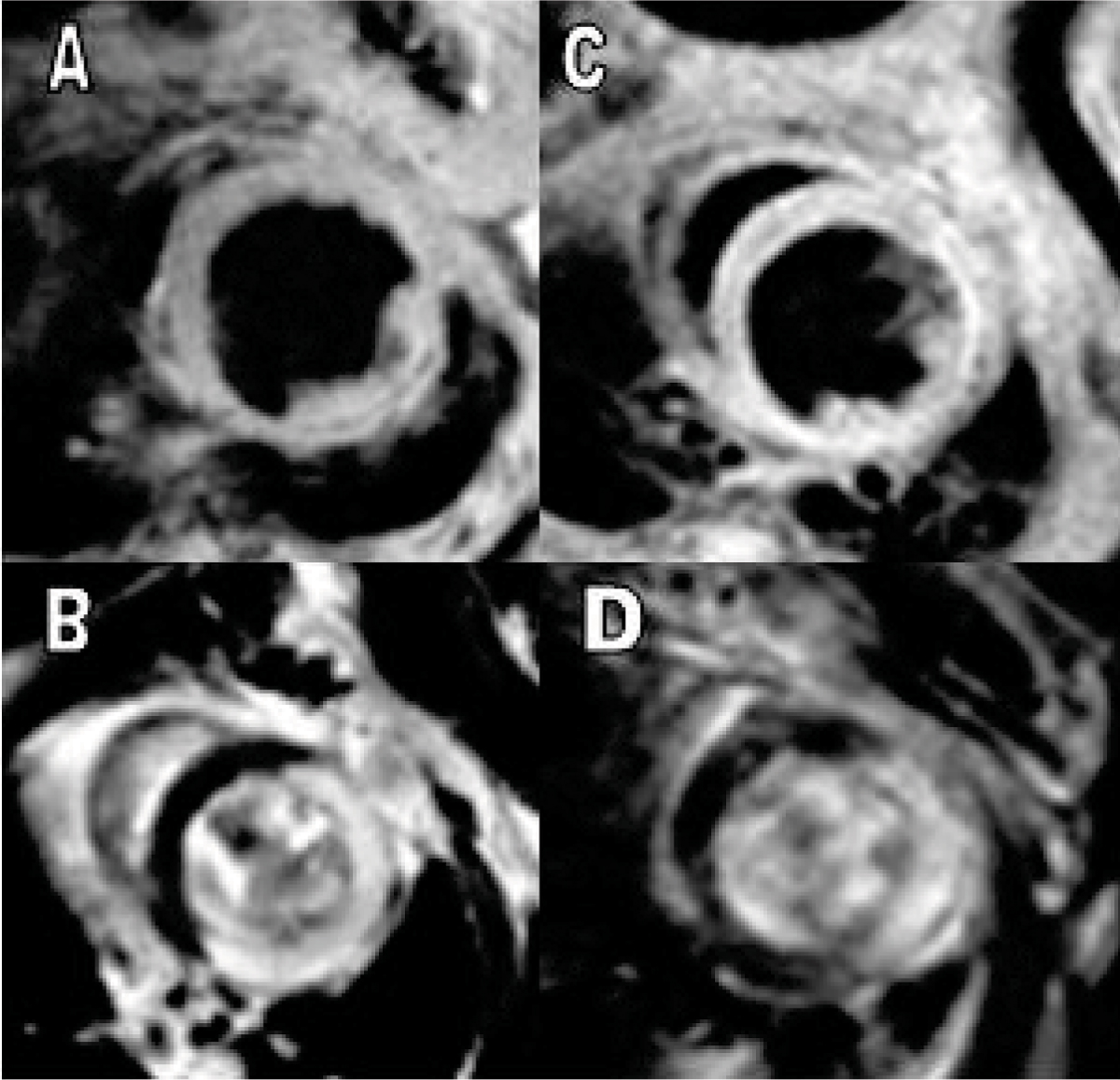
**Day 28 end systolic black blood images of C21 (A) and control (C) mice, with corresponding Day 1 DHE images shown in B and D**.

At baseline, LV end-systolic volume, mass and EF were similar between groups. Baseline LV end-diastolic volume in the treated group was larger (mean ± S.D., 48.6 ± 12.8 vs. 39.9 ± 10.6, p < 0.05). Infarct size was similar between groups (41 ± 9 vs. 38 ± 13%, p = 0.32). By CMR at day 28 post-MI, end-diastolic volume, end-systolic volume, stroke volume, and LV mass were higher in the treated group, although ejection fraction was similar. (See Table 1 and Figure 1.)

**Table 1:** Post-MI day 28 LV parameters

	LVEF (%)	LVEDV (μl)	LVESV (μl)	SV (μl)	MASS (mg)
Control (n = 17)	27.5 ± 10.5	74.1 ± 26.7	54.8 ± 24.8	19.2 ± 6.4	47.5 ± 13.9
C21 (n = 23)	27.3 ± 11.8	91.6 ± 23.8	66.9 ± 21.5	24.7 ± 13.3	56.7 ± 15.2
p value	0.98	<0.01	<0.05	<0.05	<0.05

## Conclusion

Treatment of WT C57BL/6 mice with 0.3 mg/Kg/day of the novel non-peptide angiotensin II AT_2_ receptor agonist **Compound 21** in the post-infarction period appears to cause LV hypertrophy and an increase in LV volumes without a decrement in ejection fraction. This finding counters the previously demonstrated protective benefit of transgenic overexpression of the receptor. Potential mechanisms for the lack of protective benefit of the receptor agonist include angiotensin II AT_2_ receptor down-regulation or an inadequate dose used in this preliminary study.

